# Bioactive Metabolites from the Fruiting Body and Mycelia of Newly-Isolated Oyster Mushroom and Their Effect on Smooth Muscle Contractile Activity

**DOI:** 10.3390/foods11243983

**Published:** 2022-12-09

**Authors:** Mariya Brazkova, Galena Angelova, Dasha Mihaylova, Petya Stefanova, Mina Pencheva, Vera Gledacheva, Iliyana Stefanova, Albert Krastanov

**Affiliations:** 1 Department of Biotechnology, University of Food Technologies, 26 Maritsa Blvd., 4002 Plovdiv, Bulgaria; 2 Department of Medical Physics and Biophysics, Faculty of Pharmacy, Medical University—Plovdiv, 15-A “Vasil Aprilov” Blvd., 4002 Plovdiv, Bulgaria

**Keywords:** *Pleurotus ostreatus*, bioactivity, antioxidant, biomass, basidiocarp, contractile activity

## Abstract

Higher basidiomycetes are recognized as functional foods due to their bioactive compound content, which exerts various beneficial effects on human health, and which have been used as sources for the development of natural medicines and nutraceuticals for centuries. The aim of this study was to evaluate and compare the biological potential of basidiocarp and mycelial biomass produced by submerged cultivation of a new regionally isolated oyster mushroom. The strain was identified with a high percentage of confidence (99.30%) as *Pleurotus ostreatus* and was deposited in the GenBank under accession number MW 996755. The β-glucan content in the basidiocarp and the obtained mycelial biomass was 31.66% and 12.04%, respectively. Three mycelial biomass and basidiocarp extracts were prepared, and the highest total polyphenol content (5.68 ± 0.15 mg GAE/g DW and 3.20 ± 0.04 mg GAE/g DW) was found in the water extract for both the fruiting body and the mycelium biomass. The in vitro antioxidant activity of the extracts was investigated, and it was determined that the water extracts exhibited the most potent radical scavenging activity. The potential ability of this new fungal isolate to affect the contractile activity (CA) of dissected smooth muscle preparations (SMP) was examined for the first time. It was found that oyster mushrooms likely exhibit indirect contractile effects on the gastric smooth muscle (SM) cells.

## 1. Introduction

Since prehistoric times, mushrooms have been a part of human life. It is well known that numerous ancient civilizations, including the Greek, Roman, Chinese, Mexican, Egyptian, and others, prized mushrooms as a source of food and traditional medicine. In the search for new compounds with nutritional or medicinal properties in the recent years, higher fungi of the *Agaricomycetes* class (*Basidiomycota* division) have attracted the interest of the scientific community due to their beneficial effects on human health, without negative side effects. Many representatives of *Basidiomycota* are known not only as edible, but also as medicinal fungi, and they are considered to have appealing nutritional, sensory, or health-promoting qualities.

*Pleurotus ostreatus* (Jacq.) P. Kumm. (*Basidiomycota*) is one of the most common and most studied species among oyster mushrooms. It belongs to phylum *Basidiomycota*, order *Agaricales*, family *Pleurotaceas*. Most often, solid-state cultivation is used for fruiting body formation by *P. ostreatus* using lignocellulosic side-streams from the agro-industrial production of food as a substrate [1]. Depending on the used substrate, the fruiting body formation of *P. ostreatus* could take up to 20–25 days [2]. Submerged cultivation has recently received increasing attention because it could lead to the efficient production of mycelia with uniform and reproducible qualities and valuable metabolites in a much shorter time [3].

The fruiting bodies of *P. ostreatus* have excellent organoleptic properties and high nutritional value, and mankind has consumed oyster mushrooms for thousands of years. Besides that, the metabolites derived from basidiocarps and mycelia demonstrate enviable medicinal properties [4]. These mushroom species are a significant source of a variety of bioactive compounds, such as polysaccharides, soluble and insoluble glucans, dietary fibers, proteins, polyphenols, and macro- and microelements. One of the most studied polysaccharides is the water-insoluble (1–3)-β-d-glucan called “pleuran”, obtained from *P. ostreatus* [5]. The above-mentioned bioactive substances demonstrate many medicinal activities including antibacterial [6,7], hypoglycemic [8,9], and antihyperlipidemic properties [10]. Oyster mushrooms’ metabolites can also control the immune system, suppress tumor growth and inflammation [11], lower blood cholesterol levels, prevent high blood pressure [12,13] and atherosclerosis, and perform many other functions [14].

Despite the numerous studies regarding the health-promoting properties of different mushroom extracts, the information about their effect on the contractile activity of smooth muscles is very scarce. Only three studies describe a specific action of water or alcoholic mushroom extracts on the physiological functions of smooth muscle (SM) cells: they influence 5-HT, dopamine, and acetylcholine receptor pathways and channel proteins [15], affect the contractile function of corpus SM [16], and exhibit some pharmacological effects on isolated SM [17].

The SM cells are components of the walls of hollow organs, which are part of various structures, such as the respiratory, digestive, excretory, and reproductive systems; they build up the walls of blood vessels and directly regulate their lumen [18]. The molecular mechanisms by which agonists, antagonists, therapeutics, and diseases regulate vascular SM cell contractility always occur within the context of the knowledge of whole-body function [19]. There is a wide range and variety of ion channels, ion pumps, and membrane receptors on the SM cell membrane which, on the one hand, can be affected by substances of biological or synthetic origin, and on the other hand, can affect the associated neuronal structure. This specific tissue representation allows for the characterization of a chemical or biological agent, a drug, or a natural product to be characterized as a substance with potential biological activity. A contractile or relaxant effect will show whether the examined substance affects the SM directly or indirectly.

Muscle weakness, abdominal pain, or spasm can occur in the presence of impaired contractility of gastric SM cells. All of these have a wide differential of diagnosis, non-specific symptoms, and are often neglected and difficult to influence by therapeutic intervention. The increased sensitivity to endogenous or exogenous natural or synthetic extracellular agents and the functional or morphological changes induced by them are expressed in changes in the excitability and contractility of tissues. SM cell contractile activity (CA) in normal or pathological conditions predetermines the proper functioning and motility of the gastrointestinal tract and excretory system.

Accessibility, high vitality, simplicity of the conditions in which experiments are carried out, and high sensitivity to impacting molecules or substances (concentrations in the range of 10^−9^ M) designate SM cells as preferred and reliable model systems for examining various biological and synthetic substances [20,21,22]. Through these model experiments, the effect of specific potential target molecules can be determined, thus providing opportunities for the future development of new treatment agents. The subsequent examination of the CA of bioactive compounds has aroused high interest.

In this study, fruiting bodies of a wild *Pleurotus* sp. strain were collected, and a pure culture was obtained in order to accumulate mycelium biomass through submerged cultivation. The aim of the investigation was to compare the bioactivity potential of basidiocarp and mycelium of *P. ostreatus* with regard to their glucan content and antioxidant properties and to determine, for the first time, the effect of the obtained extracts on the contractile activity of smooth muscles.

## 2. Materials and Methods

### 2.1. Materials

All reagents used in this study were of analytical grade unless otherwise stated. The organic solvents were purchased from Valerus Ltd., Sofia, Bulgaria. Rose Bengal Chloramphenicol Agar (CM0549B) was purchased from Thermo-Fisher Scientific Inc., Waltham, MA, USA; EDTA (MB011), boric acid (MB007), and Tris-base (MB029) were provided by HIMEDIA, India. The reagents Tween 80, glucose, peptone, yeast extract, and the salts were purchased from Merck KGaA, Darmstadt, Germany. All reagents were used as delivered, without further purification.

### 2.2. Fungal Isolate and In Vitro Cultivation

The fruiting bodies of the newly isolated strain were collected in Stara Planina Mountain, near Troyan, Bulgaria (42°52′23.7714″ N, 24°36’51.4836″ E) in October 2021. Healthy young representatives of the oyster mushrooms were gathered, and a purification procedure for obtaining a pure culture was performed. Briefly, the fruiting bodies were chopped into 20 ÷ 30 mm pieces after being rinsed with sterile distillate water. Following a 20 s surface disinfection with 70% ethanol and a few drops of Tween 80, they were then rinsed one more time in sterile deionized water. With a sterile scalpel, the materials were further cut into 5 by 5 mm pieces and aseptically placed onto Rose Bengal Chloramphenicol Agar (RBCA). The rest of the material was dried at 40 °C in a dryer (Pol-Eko-Aparatura, Wodzisław Śląski, Poland) and used for further experiments. The plates were incubated in darkness at 28 °C for 8 days. The resulting unidentified fungal colonies were separated and purified by repeated transferring of the developed mycelium onto fresh medium. The unknown pure fungal isolate was grown on Mushroom Complete medium (MCM), containing g/L: glucose—20.0, KH_2_PO_4_—0.5, K_2_HPO_4_—1.0, MgSO_4_—0.5, peptone—2.0, yeast extract—2.0, Agar—2.0, and pH 6.0–6.2 and was used for molecular identification.

### 2.3. DNA Extraction, PCR Amplification, Purification, and Sequencing

Prior to DNA extraction, the fungal isolate was cultivated for 8 days on MCM agar plates. The fungal mycelium was scraped out with a sterile spatula (100 ÷ 300 mg) and transferred to a 2 mL microtube. Total DNA extraction was conducted using a modified CTAB method as described by Stefanova et al. [23]. The quality and concentration of the DNA extracts were assessed by determining their absorbance at 260 nm and 280 nm (Shimadzu UV-VIS, Shimadzu Corporation, Japan).

The ITS-5.8S-ITS2 region was amplified by forward primer ITS 4 (5′-TCCTCCGCTTATTGATATGC-3′) and reverse primer ITS 5 (5′-GGAAGTAAAAGTGCTAACAAGG-3′) [24], obtained from Metabion (Martinsried, Munich, Germany). The PCR reaction mix contained 1 μL of DNA (50 ng), 0.5 μM of each primer, and 8 μL of Red-Taq DNA Polymerase Master Mix (Canvax Reagents S.L., Valladolid, Spain) in a total volume of 20 μL. The amplification was carried out in accordance with the following protocol: initial denaturation at 95 °C for 10 min, 35 cycles of 1 min at 95 °C, 1 min at 52 °C, and 1 min at 72 °C, and a final extension at 72 °C for 7 min. The amplification was carried out in a PCR 2720 Thermal Cycler (Applied Biosystems, Waltham, MA, USA). The obtained amplicon was stained with Safe View (NBS Biologicals, Huntingdon, England) and separated on 1% agarose gel carried out in 0.5× TBE buffer (45 mmol/L Trisborate and 1 mmol/L EDTA) for 50 min at 100 V, using a VWR Mini Electrophoresis system (VWR, Radnor, PA, USA) with MiniBis Pro (DNR Bio-Imaging Systems, Modi’in-Maccabim-Re’ut, Israel) for gel visualization. Finally, the PCR product was cut out from the gel and purified with a Clean-Easy™ Agarose Purification Kit (Canvax Reagents S.L., Valladolid, Spain).

The sequencing of the PCR product was performed by Microsynth Seqlab (Göttingen, Germany). The resulting sequence was analyzed using the BLAST algorithm [25] and compared to the nucleotide sequences in the GenBank database [26]. The new sequence was deposited in the GenBank database, and an accession number was assigned.

### 2.4. Submerged Cultivation for Mycelial Biomass Obtaining

Submerged cultivation of the isolate was performed in liquid MCM at 28 °C, 220 rpm for 7 days. The inoculation was made with 5% (*v*/*v*) vegetative inoculum prepared from an 8-day old culture on MCM agar slants. Each flask, containing 100 mL MCM liquid broth, was inoculated with vegetative biomass from a single MCM-slant culture. After the end of the cultivation, the biomass was separated from the cultural broth through filtration. The obtained biomass was lyophilized and used for further experiments.

### 2.5. Preparation of Extracts

The extracts of the fruiting body and the biomass were obtained with the use of ethanol (80%, *v*/*v*), methanol, and distilled water as extracting solvents. The previously dried fruiting body and the lyophilized biomass were ground using a laboratory mill (IKA, Staufen, Germany) for 30 s and mixed with 30 mL of 80% ethanol, methanol, and distilled water, respectively. The obtained samples were placed in an orbital shaker incubator (Cole-Parmer, Cambridgeshire, UK) at 25 °C and 200 rpm for 24 h. The extracts were obtained after centrifugation of the mixture. The pellet was afterwards treated with an additional 15 mL of solvent at the same conditions and centrifuged. The resulting extracts were then combined and stored at −18 °C for further investigation.

For contractile activity determination, the mushroom extracts were initially dissolved in dimethyl sulfoxide (DMSO) to prepare a 10^−2^ M stock solution. Further dilutions were made in distilled deionized water. The final concentration of the studied mushroom extracts, as well as the impacted blockers in the tissue baths, did not exceed 1:100.

### 2.6. Determination of Glucans

The quantitative determination of the contents of the total α- and β-glucans in the fruiting body and the biomass was performed using the Mushroom and Yeast β-glucan Assay Kit (Megazyme Int., Dublin, Ireland). The following procedure was applied for the estimation of the total glucan concentration in the samples: hydrolysis of the polysaccharides was performed through incubation of the biomass and the fruiting body dry samples for 2 h at 100 °C with 2 mL ice-cold 12 M sulfuric acid, followed by neutralization. The samples were then hydrolyzed to glucose by a mixture of exo-β-(1,3)-D-glucanase plus β-glucosidase in sodium acetate buffer (pH 4.5) for 1 h of incubation at 40 °C. An enzymatic hydrolysis with amyloglucosidase and invertase was conducted for α-glucan content estimation. With the addition of glucose oxidase/peroxidase reagent and measuring the absorbance at 510 nm, the total and α-glucan content were determined. The calculation of the β-glucan content was made by subtracting the α-glucan from the total glucan content. All values of total α- and β-glucans in the biomass were expressed as g/100 g of a DW biomass.

### 2.7. Determination of Total Phenolic Content (TPC)

The TPC was analyzed following the method of Kujala et al. [27], with some modifications. Each extract (0.1 mL) was mixed with 0.5 mL Folin–Ciocalteu reagent and 0.4 mL 7.5% Na_2_CO_3_. The mixture was vortexed and left for 5 min at 50 °C. After incubation, the absorbance was measured at 765 nm. The TPC is expressed as mg gallic acid equivalents (GAEs) per gram dry weight (g DW).

### 2.8. Determination of In Vitro Antioxidant Activity

#### 2.8.1. DPPH^•^ Radical Scavenging Assay

The ability of the extracts to donate an electron and scavenge 2,2-diphenil-1-picrylhydrazyl (DPPH) radicals was determined by the method described by Angelova et al. [28]. The DPPH radical scavenging activity is presented as TEAC (Trolox equivalent antioxidant capacity)—a function of the concentration of Trolox—and is defined as the concentration of Trolox with equivalent antioxidant activity expressed as μM TE/g DW.

#### 2.8.2. ABTS^•+^ Radical Scavenging Assay

The radical scavenging activity of the extracts against 2,2′-azino-bis(3-ethylbenzothiazoline-6-sulfonic acid) (ABTS^•+^) was estimated according to the method described by Angelova et al. [28], and the results are expressed as a TEAC value (μM TE/g DW).

#### 2.8.3. Ferric-Reducing Antioxidant Power (FRAP) Assay

The FRAP assay was carried out according to the procedure of Angelova et al. [28]. The FRAP reagent was fresh, prepared daily, and was warmed to 37 °C prior to use. The absorbance was recorded at 593 nm, and the results are expressed as TEAC, μM TE/g DW.

#### 2.8.4. Cupric Ion-Reducing Antioxidant Capacity (CUPRAC) Assay

The CUPRAC assay was carried out according to the procedure described by Angelova et al. [28]. Trolox was used as a standard, and the results are expressed as μM TE/g DW (TEAC).

### 2.9. Contractile Activity

#### 2.9.1. Tissue Preparation

All procedures were approved by the Institutional Animal Care and Use Committee of Bulgaria and complied with the EU Directive 2010/63/EU.

Male Wistar rats with body weights in the range of 250 ÷ 275 g were used. One animal per day was used for the experiments, using three organ baths simultaneously, measuring three to five muscle strips per animal. The gastric smooth muscle preparations (SMPs) cut in the direction of the circular muscle layer from the rat stomach were rinsed immediately in pre-gassed (95% O_2_, 5% CO_2_) KH at 4 °C until further processing. The number of SMPs used for each data point is indicated by *n*. All efforts were made to minimize the number of animals used, as well as their suffering.

#### 2.9.2. Measurement of Contraction in Smooth Muscle Strips

The SM strips with 12 ÷ 13 mm in length and 1.0 ÷ 1.1 mm in width were mounted vertically in a 20 mL tissue bath containing oxygenated (95% O_2_/5% CO_2_) with Kreb’s medium (pH 7.4) at 37 °C and were attached to a stationary glass holder at one end and to Swema tensodetectors (Stockholm, Sweden) at the other. The signal of the tensodetectors presenting the SM tissue CA was amplified by K. Tesar—D 486 amplifier (Selb, Germany) or Microtechna amplifier (Prague, Czech Republic). The mechanical activity was recorded on paper using a Linseis polygraph recorder (Selb, Germany).

The value of the initial mechanical tension of the preparations, obtained by stretching the tension system, corresponded to a tension force of 10 mN. For the purposes of stabilizing the muscle tonus and spontaneous CA, about 60 min were allowed to elapse, during which time the Krebs solution in the tissue bath was changed 2 ÷ 3 times. After a contractile response was monitored, tissues were washed and allowed to return to the baseline tension in fresh buffer for 35 min (adaptation period). Treatment of the individual muscle strips was chosen depending on the vitality of the muscle tissue. The baseline tonus value following this adaptation period was accepted as the zero value. A control contraction was elicited by applying acetylcholine (ACh, 1 μM) to the tissue bath solution. Muscle strips not showing spontaneous contraction were discarded and replaced. Measurement began when spontaneous muscle contractions occurred frequently for at least 2 min. The substance-induced alterations (contractions) were recorded as a positive change regarding this value. Frequency, mean peak height, and the intensity of the peak were detected. Muscle contraction was then stimulated using the mushroom extracts alone and in the presence of Ca^2+^ blockers (nifedipine, verapamil, and atropine). Control experiments in the presence of equal concentrations of the vehicle DMSO showed no significant effect on ACh contraction.

### 2.10. Statistical Analysis

All experiments were conducted in triplicate, and the values were expressed as mean ± SD. Statistical significance was determined by analysis of variance (ANOVA, Tukey’s test; the value of *p* < 0.05 indicated statistical difference).

## 3. Results and Discussion

### 3.1. Identification of the Fungal Isolate

Newly formed fruiting bodies of wild *Pleurotus* sp. were collected in Stara Planina Mountain in Bulgaria, and the samples were processed. Molecular identification of the pure culture was performed by the amplification of the ITS1-5.8S-ITS2 region, and the obtained PCR product was subjected to sequence analysis. The resulting sequence was analyzed using the BLAST algorithm and compared to the nucleotide sequences in the GenBank database [26]. The strain was identified as *P. ostreatus*, with 99.30% percent confidence. The ITS1-5.8S-ITS2 rRNA gene sequence of *P. ostreatus* GA2M was deposited in the GenBank under accession number MW 996755.

The oyster mushrooms belonging to the genus *Pleurotus* (*Pleurotaceae* family) are often found in Bulgaria and are easily recognizable by their specific manner of growth on wood in the form of shelf-like clusters. They appear on living or dead rotting trees, with a preference for deciduous rather than coniferous species, between October and April. In Bulgaria, *Pleurotus ostreatus*, *Pleurotus pulmonarius*, and *Pleurotus cornucopiae* are commonly found. It is not always easy to distinguish the closely related species, although *P. ostreatus* is not able to mate with other species [29].

### 3.2. Submerged Cultivation of P. ostreatus GA2M for Obtaining Biomass

The cultivation of *P. ostreatus* GA2M was performed in submerged culture for 7 days in liquid MCM medium. The strain possesses a pellet growth pattern, and 12.98 ± 0.59 g dry mycelium biomass per liter of medium were obtained. Vamanu [30] reported much higher mycelium yields (nearly 40 g DW/L) when cultivating *P. ostreatus* strains in a bioreactor. The ability to maintain constant cultivation conditions, such as temperature, pH, aeration, and stirring, along with an optimized medium composition, could lead to greater than 20% higher yields of mycelium biomass.

A comparison between the wild fruiting body and the obtained mycelial biomass was made with regard to glucan content, antioxidant activity, and CA.

### 3.3. Determination of Glucans

Glucan content determination was performed with lyophilized dry samples, and the results are presented in Table 1.

The obtained data clearly shows the predominance of glucan content in the fruiting body sample, with a total amount of 35.84 ± 4.16% and only 12.28 ± 1.24% in the mycelial biomass. The β-glucan concentration in both samples prevails over the one with the α-glucans. Oyster mushrooms are a major β-glucan source, and variations in the concentrations of the total α- and β-glucans in the different species are common. For example, the total glucan content could vary from 18.26% to 25.64%, and the β-glucan concentration ranges from 15.3% to 24.4% [31]. The calculated *p*-values indicate a statistically significant difference between the glucan concentration in the fruiting body and the mycelium, which is in agreement with results from previous studies [32]. Despite their confirmed anti-neoplastic, anti-oxidative, and immunomodulatory properties, α-glucans are not an object of intense investigation. Their concentration in various species of oyster mushrooms varies in the range 2.0 ÷ 4.0%, and it is usually lower in comparison with β-glucan concentrations [31].

Mushroom polysaccharides have great commercial importance since they possess a number of beneficial properties. Moreover, those polysaccharides have an important structural role, representing about 60% of the dry weight of the cell walls [33]. They could be found in the fruiting bodies, as well as in the mycelium biomass of the mushrooms. Many factors, such as species, cultivation conditions, and age, could affect the glucan concentration in the fungi. In particular, *P. ostreatus*, being one of the widely cultivated mushrooms, attracts the interest of the scientific community because the glucans in their composition are proven to have immunomodulatory, antioxidant, anti-inflammatory, and analgesic properties [34].

Although the fruiting bodies of the mushroom have higher glucan content, with the employment of correct methodology and cultivation conditions, the mycelial biomass could become a very valuable source of glucans. Moreover, the submerged fungal cultures are preferable due to the easy polysaccharide preparation compared to the laborious manual harvesting. Studies have demonstrated no significant difference between the biological activities of the glucans isolated from the mycelia or the fruiting bodies of *P. ostreatus* [35].

### 3.4. Determination of TPC and In Vitro Antioxidant Activity

Primary and secondary metabolites contained in mushrooms exhibit beneficial effects, including antioxidant activity. Antioxidant compounds have been extracted from the fruiting bodies, mycelium, and broth of various mushrooms [36]. To assess the antioxidant potential of the newly isolated *P. ostreatus* strain, three extraction procedures were carried out separately with both mycelial biomass and basidiocarp. Commonly used solvents—water, ethanol, and methanol—reported to be suitable for mushroom extraction [37,38,39,40], were used to recover most of the bioactive substances.

The first step was to estimate the TPC in the samples. TPC varied between 0.21 ± 0.01 and 5.68 ± 0.15 mg GAE/g DW (Table 2). The TPC content in the dry biomass of *P. ostreatus* was significantly lower compared to that from the fruiting body. Furthermore, regardless of the solvent, the fruiting body contained more TPC, but still the predominant amount was extracted with water as a solvent. The calculated *p*-values indicate that the difference between the TPC content in the fruiting body and the mycelia is of statistical significance. Similar results regarding the total phenolic content of the fruiting bodies and submerged cultured mycelium of Indian *P. ostreatus* were reported by Prasad et al. [41]. 

Regarding antioxidant activity, the trend was similar. The biomass had less potential than the fruiting body (Table 2), and this trend was confirmed by the performed statistical analysis of the data. This is consistent with the data reported for the medicinal mushroom *Trametes versicolor* [28,42]. According to the two antiradical assays (DPPH and ABTS), the values for mycelial biomass activity were below the limit of quantification. However, the four assays applied in the evaluation of the antioxidant potential showed an unequivocal preponderance of all fruiting body extracts, especially for the aqueous extract.

Fruiting body extract had a better ability to reduce the DPPH free radical (IC_50_, 0.45 ± 0.04 mg/mL), with the total phenolic compound of the fruiting body extract being higher (4.62 ± 0.08 mg GAE/g extract) than that of the mycelia extract [43].

However, some authors obtained contradictory results, and in particular, fruiting body extracts were more effective in DPPH radical scavenging activity and lipid peroxidation inhibition than mycelial extracts, but the mycelium extracts were more effective in terms of ABTS radical scavenging activity and iron ion chelating ability [44].

It is common that the fruiting bodies and the mycelium of the culture of all mushroom species show different antioxidant capacity [41].

The wide range of the established antioxidant potential may be due to the different compounds present in the extracts. Moreover, AOA is not only attributed to polyphenols, but different types of polyphenols also possess quite different reactivity, and furthermore, synergistic and antagonistic effects are possible. Still, water as a solvent proved to be advisable and in full agreement with the green concept and the need for sustainability [45].

The trend of TPC and AOA results showed a relationship that was consistent with the positive correlation between polyphenol content and antioxidant potential reported by Chun et al. [46] and Angelova et al. [28]. In addition, basidiocarp β-glucan content (31.66 ± 3.98%) was predominant, revealing a correlation between TPC, AOA, and basidiocarp β-glucan content. β-glucan exerts effects on the activation of the immune system, as well as antimicrobial, antioxidant, antiviral, antifungal, antitumor, cholesterol-lowering, and blood sugar-regulating effects [47].

In summary, the fruiting body of *P. ostreatus* can be considered a source of natural antioxidants. This fact is in agreement with the recommendation of consumption of both the fruiting body and the mycelial mass of *A. brasiliensis* as beneficial for health [44].

### 3.5. Contractile Activity Determination

In living organisms, β-glucans stimulate and modulate the action of macrophages and are, therefore, considered biological response modifiers. Polyphenols and antioxidants have also been shown to modulate biological activity, both at the level of the organism and in isolated tissues [36].

**Figure 1:**
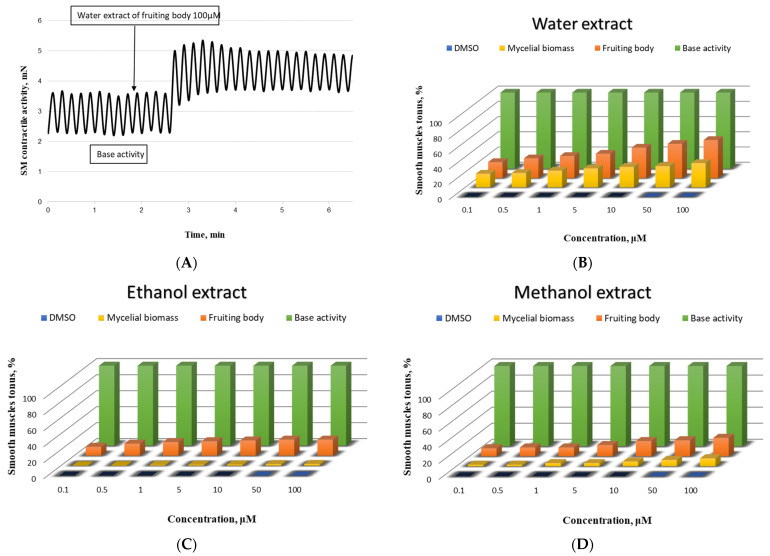
Biological activity of the newly isolated *Pleurotus ostreatus* strain on the SMPs. (**A**)—Representative tracing of gastric SM elicited by 100 μM of the applied mushroom extract of the fruiting body; concentration-effect dependence of the effect of mushroom sample (water extract—(**B**), ethanol extract—(**C**), and methanol extract—(**D**)) on the muscle tonus of SM from the stomach (*n* = 9); base activity is taken as 100%.

Since the results obtained for the β-glucan and the TPC and antioxidant activity of the fruiting body and mycelial biomass were quite promising, the benefit of their effect on the contractile activity of SMPs was investigated. In our ex vivo experiments, the studied mushroom extracts rich in β-glucan and TPC (Table 1 and Table 2) demonstrated a dose-dependent contractile effect comparable to that of natural products already scientifically reported for other representatives of this class [15,17]. The results are illustrated in Figure 1.

The most pronounced contractile effect was observed in SM cells incubated with water extract from a fruiting body (Figure 1A). The changes in the CA were also significant after the incubation with water extract of mycelial biomass applied in an equivalent concentration (Figure 1B). In the experiments with the methanol extract, a higher value was again recorded for the fruiting body, compared to that of the mycelial biomass (Figure 1D), while for the ethanol extracts of both samples, no statistically significant difference was found in the shortening activity of the treated compared to the auto-control SMPs in the entire concentration range (Figure 1C).

The analysis of the obtained ex vivo results leads us to the conclusion that the clarification of the specific ways of the investigated mushroom extract impact on the contractility of the SM cells is justified only for the aqueous extracts of the fruiting body, in full accordance with the most pronounced in vitro results for these extracts in the present work.

In the studies of Kumakura et al. [48], water extract from the fruiting body demonstrated the properties of a functional molecule. Numerous other studies confirm the biological activity of the compounds found in these extracts because of the presence of β-glucans, polyphenols, and antioxidants.

Phenolic compounds are antioxidants with redox properties that allow them to act as reducing agents, hydrogen donors, free radical scavengers, and singlet oxygen quenchers [49]. Cells respond to these molecules mainly by direct interactions with receptors or enzymes involved in signal transduction, leading to modification of the redox status of the cell and triggering a series of redox-dependent reactions [50,51]. Presumably, the moderate contractile effects are due to the high concentration of polyphenols and β-glucans, which inhibit the growth of pathogenic bacteria such as *E. coli*, *Clostridium*, and *Salmonella* [52], thus improving the immunity of the gastrointestinal tract through their prebiotic activity. In addition to antioxidant capacity, most of these compounds have several different molecular targets, act on several signaling pathways, and induce pleiotropic and neuroprotective effects [53] in cells.

The water extract of the fruiting body demonstrated biological activity. In our experiments, it was expressed in SM tissue contraction that increased in strength with the increase in the extract concentration in the tissue bath. As the main cause for the observed effect, we accept the ability of the investigated substance to indirectly increase the level of cytosolic Ca^2+^, which is mainly responsible for SM cell contractions. According to classical concepts, one of the main pathways for increasing the intracellular Ca^2+^ level is the entry of Ca^2+^ ions from the extracellular space through membrane Ca^2+^ channels [54,55]. When they are blocked by specific or non-specific blockers, SM contractility decreases [56]. It is probable that the mentioned processes are affected in our studies because, in conditions of pre-applied Ca^2+^ channel blockers (atropine, verapamil, or nifedipine) in the tissue bath, we registered a significant reduction or complete lack of contractile response upon subsequent exposure to the fruiting body aqueous extract (Table 3). This result indicates the participation of the investigated mushroom extracts in the modulation of the Ca^2+^ transmembrane concentration gradient and defines its role as a biologically active substance.

Gastric SM cell contractions support the optimal functioning of the digestive system. The frequency, amplitude, and tone of spontaneous contractions can be influenced and corrected by a number of hormones, neurotransmitters, receptor, and channel activators and blockers, drugs, and exogenous substances rich in β-glucans, dietary fiber, and antioxidants. Therefore, the study of the activation-contraction relationship in the mushroom extract-SM model system is interesting from the point of view of a potential clinical factor.

## 4. Conclusions

This is the first study report of a promising dose-dependent contractile effect on the smooth muscle cells of an aqueous extract obtained by newly isolated and molecularly identified *Pleurotus ostreatus*. It could be hypothesized that the established high β-glucan and TPC content of aqueous extracts, along with significant antioxidant activity, are related to the demonstrated contractile activity. Further research is required to clarify in detail the relationship between the compounds in the mushroom extract and the frequency, amplitude, and tone of spontaneous contractions of the gastric SM cells in order to develop new natural medicinal products or dietary supplements supporting the optimal functioning of the digestive system.

## Figures and Tables

**Table 1:** Glucan content (%) in the fruiting body and mycelial biomass of *Pleurotus ostreatus*.

Fruiting Body			Mycelial Biomass		
Total glucans	α-glucans	β-glucans	Total glucans	α-glucans	β-glucans
35.84 ± 4.16	4.18 ± 0.59	31.66 ± 3.98	12.28 ± 1.24	0.282 ± 0.047	12.04 ± 1.85

**Table 2:** Total phenolic content (TPC, mg GAE/g DW) and antioxidant potential (CUPRAC, FRAP, ABTS, and DPPH assays, μM TE/g DW) of newly isolated *Pleurotus ostreatus* strain extracts.

Sample		TPC	CUPRAC	FRAP	ABTS	DPPH
Water extract	Mycelial biomass	3.20 ± 0.04	16.63 ± 0.29	11.08 ± 0.07	under LOQ *	under LOQ
	Fruiting body	5.68 ± 0.15	24.30 ± 0.44	12.02 ± 0.30	11.36 ± 0.57	3.50 ± 0.73
Ethanol extract	Mycelial biomass	0.21 ± 0.01	11.01 ± 0.62	3.27 ± 0.06	under LOQ	under LOQ
	Fruiting body	2.01 ± 0.02	22.51 ± 0.30	7.22 ± 0.05	7.51 ± 1.04	2.89 ± 0.07
Methanol extract	Mycelial biomass	1.08 ± 0.06	16.41 ± 0.80	2.08 ± 0.07	under LOQ	under LOQ
	Fruiting body	1.81 ± 0.02	19.22 ± 0.91	3.33 ± 0.07	10.26 ± 0.36	2.21 ± 0.05

* under LOQ—under limit of quantification.

**Table 3:** Changes in the tonic activity of the SMPs incubated with water extract of the fruiting body at 100 μM concentration after application of Ca^2+^ channel blockers, * *p* < 0.05.

Tonic Activity of Water Extract of Fruiting Body 100 μM				
Initial Reaction, mN	Applied Blocker	Resultant Reaction, mN	*n*	*p*
1.38 ± 0.09	0.5 μM nifedipine	0 *	9	0.002
1.35 ± 0.15	0.3 μM verapamil	0.52 ± 0.02 *	12	0.003
1.42 ± 0.05	1 μM atropine	0.27 ± 0.05 *	12	0.002

## Data Availability

Data is contained within the article.
